# Trade‐off between early emergence and herbivore susceptibility mediates exotic success in an experimental California plant community

**DOI:** 10.1002/ece3.2610

**Published:** 2016-11-30

**Authors:** Joseph Waterton, Elsa E. Cleland

**Affiliations:** ^1^ Ecology, Behavior and Evolution SectionUniversity of California San DiegoLa JollaCAUSA

**Keywords:** community assembly, emergence, germination, herbivory, invasion, phenology, seedlings

## Abstract

Ecological trade‐offs are fundamental to theory in community ecology; critical for understanding species coexistence in diverse plant communities, as well as the evolution of diverse life‐history strategies. Invasions by exotic species can provide insights into the importance of trade‐offs in community assembly, because the ecological strategies of invading species often differ from those present in the native species pool. Exotic annual species have invaded many Mediterranean‐climate areas around the globe, and often germinate and emerge earlier in the growing season than native species. Early‐season growth can enable exotic annual species to preempt space and resources, competitively suppressing later‐emerging native species; however, early‐emerging individuals may also be more apparent to herbivores. This suggests a potential trade‐off between seasonal phenology and susceptibility to herbivory. To evaluate this hypothesis, we monitored the emergence and growth of 12 focal species (six each native and exotic) in monoculture and polyculture, while experimentally excluding generalist herbivores both early and later in the growing season. Consistent with past studies, the exotic species emerged earlier than native species. Regardless of species origin, earlier‐emerging species achieved greater biomass by the end of the experiment, but were more negatively impacted by herbivory, particularly in the early part of the growing season. This greater impact of early‐season herbivory on early‐active species led to a reduction in the competitive advantage of exotic species growing in polyculture, and improved the performance of later‐emerging natives. Such a trade‐off between early growth and susceptibility to herbivores could be an important force in community assembly in seasonal herbaceous‐dominated ecosystems. These results also show how herbivore exclusion favors early‐active exotic species in this system, with important implications for management in many areas invaded by early‐active exotic species.

## 1 Introduction

Ecosystems with Mediterranean‐type climates harbor exceptional plant diversity and are among the most at risk to biodiversity loss in the coming decades due to multiple factors including changes in climate and land use, as well as invasion by exotic species (Sala et al., 2000; Underwood, Viers, Klausmeyer, Cox, & Shaw, 2009). Exotic species often differ from native species in suites of functional traits related to strategies for resource capture (Godoy, Valladares, & Castro‐Díez, 2011; van Kleunen, Weber, & Fischer, 2010; Leishman, Haslehurst, Ares, & Baruch, 2007). In the Mediterranean‐climate regions of California and southwestern Australia, where the onset of the growing season is initiated by the start of winter rains, exotic annual species have been observed to emerge faster and earlier than native species (Abraham, Corbin, & D'Antonio, 2009; Deering & Young, 2006; Pérez‐Fernández, Lamont, Marwick, & Lamont, 2000; Reynolds, Corbin, & D'Antonio, 2001; Wainwright & Cleland, 2013; Wainwright, Wolkovich, & Cleland, 2012). Early emergence allows exotic species to preempt resources and competitively suppress the growth of later‐active natives, a type of seasonal priority effect (Wainwright et al., 2012). Priority effects mediated by such differences in arrival time have been shown to exert strong effects on community structure and function (Fukami, 2015). This is demonstrated by experiments in which the arrival time of late‐germinating native species is experimentally advanced, leading to competitive suppression of later‐planted exotic species (Cleland, Esch, & McKinney, 2015; Grman & Suding, 2010), with these effects sometimes persisting over multiple years (Vaughn & Young, 2015). The results of such experiments raise an important question: Why do certain species, including many natives, display later emergence within the growing season despite the competitive advantage associated with earlier activity? Selection for early phenology has been hypothesized to be limited by exposure to stressful abiotic conditions before the onset of consistent growing season conditions (e.g., Anderson, Inouye, McKinney, Colautti, & Mitchell‐Olds, 2012; Augspurger, 2013), and could also expose early‐active individuals to a greater risk of herbivory (Hanley, 1998).

Exclusion experiments have shown that herbivores, particularly small mammals, exert strong control over plant productivity in herbaceous communities in Mediterranean‐climate regions (Peters, 2007). In these ecosystems, there is often little herbaceous vegetation suitable for consumption prior to the arrival of seasonal rains, meaning individuals that emerge first may initially represent the only herbaceous vegetation available to consumers and are also likely to be highly apparent and accessible in the landscape (Wainwright et al., 2012). Furthermore, individuals that emerge earlier generally grow to be larger, through longer growing time and seasonal priority effects, and as a result may be selectively targeted even after the initial period following emergence (Hulme, 1994). In contrast, seedlings emerging later might benefit from association with larger established neighbors if this makes them less apparent and accessible, a form of associational resistance (Underwood, Inouye, & Hambäck, 2014).

Herbivory during the earliest period of the growing season is likely to be particularly impactful for newly emerging individuals because seedlings tend to have weak structural defenses and low concentrations of some defense compounds, and are often of greater nutritional quality than mature plants (Quintero, Lampert, & Bowers, 2014). Seedlings are also small relative to the size of consumers; hence, herbivory is often fatal (Fenner & Thompson, 2005). For example, mortality in six California grassland species exposed to gastropods has been shown to be greater in the first three weeks following emergence than the following 2 months combined (Cleland, Peters, Mooney, & Field, 2006). For seedlings that survive attack, stored reserves and photosynthetic capacity are lower in these early ontogenetic stages; as such, tolerance to herbivory, the ability to regrow and reproduce following damage, is generally low (Boege & Marquis, 2005; Strauss & Agrawal, 1999). Therefore, any trade‐off between emergence time and susceptibility to herbivores is likely to be most apparent when herbivores are active in the earliest period of the growing season. Furthermore, such large impacts of herbivory in the early season may lead to large effects on community‐level productivity; however, such effects of the timing of herbivory within a growing season have largely been overlooked (but see Sullivan & Howe, 2009).

Release from specialist enemies and avoidance by generalist herbivores in introduced ranges are two mechanisms often hypothesized to promote invasion by exotic species (i.e., the enemy release hypothesis; Keane & Crawley, 2002). However, generalist mammalian herbivory has been shown in many cases to slow the invasion of exotic herbaceous species into Mediterranean‐climate regions (Cushman, Lortie, & Christian, 2011; Lambrinos, 2006; Peters, 2007; Rice, 1987; Vilà & D'Antonio, 1998; Wainwright et al., 2012). Evolutionary naivete due to a lack of coevolution with herbivores is often cited as an explanation for such high impacts of herbivory on exotic species (Colautti, Ricciardi, Grigorovich, & MacIsaac, 2004; Parker, Burkepile, & Hay, 2006); yet, our understanding of the particular plant traits underlying herbivore preference for exotic species is limited. If earlier emergence per se leads to greater overall susceptibility to herbivores, this could provide a mechanistic explanation for why many exotic species appear to be heavily impacted by generalist herbivores.

Previous studies investigating the mechanisms underlying the impacts of generalist herbivores on community assembly have largely focused on the role of differences in plant defense. In such studies, germination of focal species is often staggered to ensure synchronous emergence (e.g., Burt‐Smith, Grime, & Tilman, 2003) or plants are presented to herbivores simultaneously (e.g., Hanley & Sykes, 2009; Kempel et al., 2015). However, such studies are unlikely to represent the action of herbivores in nature when emergence time varies greatly between species. Furthermore, much of our understanding of herbivore impacts on community assembly comes from studies on invertebrates (reviewed in Barton & Hanley, 2013); however, mammals consume a higher proportion of tissue of plants they encounter (Hulme, 1994) and can exert at least as great an impact on herbaceous production in Mediterranean ecosystems as invertebrate herbivores (e.g., Peters, 2007).

This study tested two hypotheses regarding the relationship between emergence timing and mammalian herbivory on species performance: (1) earlier‐emerging species are more susceptible to herbivores, and this susceptibility is greatest early in the growing season; and (2) in polyculture, the competitive advantage of early‐arriving exotic species is reduced by herbivory, and this effect is greatest early in the growing season.

## 2 Methods

### 2.1 Study site

The experiment was conducted at the University of California, San Diego Biology Field Station from November 2014 to January 2015. This site is characterized by a flat, regularly tilled experimental field with sandy clay loam soil. Prior to planting focal species, the experimental site was dominated by exotic species including *Hordeum murinum* (Poaceae), *Erodium cicutarium* (Geraniaceae), and *Malva parviflora* (Malvaceae). The site is inhabited by two native generalist mammalian herbivores: the brush rabbit (*Sylvilagus bachmani*) and desert cottontail (*Sylvilagus audubonii*). However, no data are available on the density of these two herbivore species during the experiment or at other times.

### 2.2 Experimental design

Twelve focal species (Table 1), six native and six exotic, were used to test our two hypotheses. These locally common native and exotic species were taxonomically balanced at the family level, with six species in the families Fabaceae and Poaceae. Additional two species, *Rumex crispus* and *R. salicifolius* (Polygonaceae), were also used; however, these failed to emerge and were thus excluded from all analyses. Seeds were obtained from a commercial supplier (S&S Seeds, Carpinteria, California). None of the focal species used in the experiment were observed growing in the area prior to the experiment, assuring that all individuals measured did not emerge from an existing seed bank.

**Table 1:** Focal species used in the experiment, along with abbreviations used in figures, origin (with reference to California), family, and life history

Scientific Name	Abbreviation	Origin	Family	Life History
*Acmispon americanus*	ACM AM	Native	Fabaceae	Annual
*Lupinus bicolor*	LUP BIC	Native	Fabaceae	Annual
*Trifolium willdenovii*	TRI WIL	Native	Fabaceae	Annual
*Bromus carinatus*	BRO CAR	Native	Poaceae	Perennial
*Festuca microstachys*	FES MIC	Native	Poaceae	Annual
*Festuca rubra*	FES RUB	Native	Poaceae	Perennial
*Medicago polymorpha*	MED POL	Exotic	Fabaceae	Annual
*Trifolium hirtum*	TRI HIR	Exotic	Fabaceae	Annual
*Vicia villosa*	VIC VIL	Exotic	Fabaceae	Annual
*Bromus hordeaceus*	BRO HOR	Exotic	Poaceae	Annual
*Festuca myuros*	FES MY	Exotic	Poaceae	Annual
*Festuca perennis*	FES PER	Exotic	Poaceae	Annual/Perennial

Plants were grown under one of three herbivore treatments: early exclusion, late exclusion, and no exclusion. Herbivore exclosures were constructed using 6.3‐mm hardware cloth, designed to exclude all vertebrate, but not invertebrate herbivores. Measurements of photosynthetically active radiation (PAR) were taken with an AccuPAR LP‐80 PAR Ceptometer (Decagon Devices Inc., Washington, USA); shading by exclosures reduced PAR by approximately 30% (Table S1 in Supporting Information). Herbivores were excluded for the first half of the experiment in the early exclusion treatment, the second half of the experiment in the late exclusion treatment, and were allowed access for the entire duration in the no exclusion treatment.

**Figure 1:**
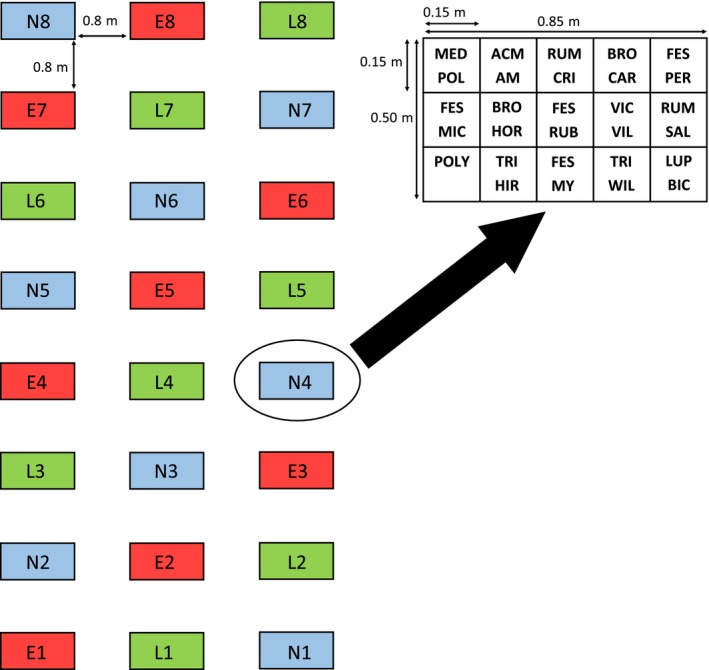
Experimental design showing the arrangement of 24 plots (left) and example arrangement of subplots within a single plot (above). One plot in each of early, late, and no exclusion treatments (red, green, and blue rectangles, respectively) is represented in every block of three plots. Within plots, one polyculture subplot (POLY) and 14 monoculture subplots (see Table 1 for species abbreviations) are arranged in a 3 × 5 grid. Two monoculture subplots were planted with *Rumex* spp. (“RUM CRI” and “RUM SAL”); these failed to emerge and were removed from all analyses

Each species was grown in both monocultures and in polycultures containing all 12 species. Eight plots were assigned to each herbivore exclusion treatment, with each plot containing 12 monoculture subplots and one polyculture subplot (each subplot was each 15 cm × 15 cm with 2.5 cm between adjacent subplots), for a total of 288 monoculture and 24 polyculture subplots (Figure 1). When in place, single‐herbivore exclosures covered entire plots. To account for effects of subplot location within plots, eight planting plans that mixed the edge versus interior positions of subplots within plots were used for each exclusion treatment. Plots were spaced 0.8 m apart and arranged in a 3 × 8 grid with one plot of each exclusion treatment in every row of three (Figure 1). Rows were arranged at different distances from nearby greenhouses (which might potentially affect herbivore activity), so these are hereafter referred to as block for statistical analyses. Monoculture subplots were planted with seeds to achieve 14 emergents, and polyculture subplots were planted with seeds to achieve two emergents of each species (Table S2 contains emergence rates and numbers of seeds planted for each focal species). While these densities are lower than those previously reported in California grasslands (Bartolome, 1979; Heady, 1958), they were chosen to ensure the emergence of individual plants could be accurately monitored. Greenhouse emergence trials were conducted prior to the start of the experiment to estimate the number of seeds required to produce the targeted number of individuals in monoculture and polyculture, and to aid identification of emergents in the field.

The experimental area was watered and raked daily for 2 weeks before the start of the experiment to emerge and remove the standing seed bank. Seeds of focal species were planted on 3rd November, 2 days after the first winter rain event. To stimulate emergence, 1 L of water was applied evenly across each plot on a daily basis from 8th November until the next rain event on 20th November. Weeds were removed by hand throughout the experiment. The number of surviving emergents and cumulative total number of emergents were monitored daily in monoculture subplots beginning 9th November when the first emergent was observed; this was designated as Day 1 of the experiment. Due to the difficulty of seedling identification, emergence was not monitored in polyculture subplots.

Newly emerged individuals were recorded for 35 days (until 13th December), at which point exclosures were transferred from early exclusion treatments to late exclusion treatments. Beginning 16th January, 35 days after transferring exclosures and 70 days after the first emergence event was observed, all remaining individuals were counted and aboveground biomass was harvested. The length of the experiment was aimed to match the length of time between widespread emergence and peak biomass observed in local herbaceous‐dominated vegetation communities, estimated at 2.5 months (see Figure 22.5 in Cleland, Funk, & Allen, 2016). No plants of any species had begun flowering at the time of harvest. To confirm that the majority of emergence had occurred by the midway point of the experiment, the difference in the mean number of individuals harvested in each subplot after 70 days and the mean cumulative total of emergents observed after 35 days (regardless of mortality) was calculated for each species. This value was below one for all species, confirming that most emergence had occurred by this point.

Emergence time was calculated as the number of days taken to reach 50% of total emergence after 35 days. The number of individual plants at time of harvest was used as a measure of density in monoculture and polyculture subplots. Harvested plants were dried at 40°C for 7 days, and total biomass in monoculture subplots and biomass of each species in polyculture subplots measured.

To assess the performance of focal species in polyculture under each exclusion treatment (hereafter referred to as “relative performance in polyculture” [RPP]), we calculated the proportional deviation of a species’ yield in polyculture from its yield in monoculture in the same exclusion treatment (sensu Loreau, 1998). The mean biomass of individual plants of a species in monoculture and polyculture, pooled across all eight plots within exclusion treatments, were used as measures of yield to calculate RPP, as given by the following equation:

$$\text{RPP}=\frac{P-M}{M}$$

where *P* and *M* represent the mean biomass per individual of a species in a particular exclusion treatment in polyculture and monoculture, respectively. An RPP value of 0 indicates no difference in performance (individual size) between monoculture and polyculture for a species in a particular exclusion treatment. An RPP value below 0 indicates plants grown in polyculture were on average smaller than those in monoculture, while an RPP value greater than 0 indicates plants grown in polyculture were on average larger than those in monoculture.

### 2.3 Statistical analyses

Statistical analyses were conducted using R version 3.2.3 (R Core Team 2015). Linear mixed‐effects models were specified with the *lme* function in the package *nlme* (Pinheiro, Bates, DebRoy, & Sarkar, 2015). Linear fixed‐effect models were specified with the *lm* function in the package *stats*. Significance of all factors was evaluated with Type II tests using the *Anova* function in the *car* package (Fox & Weisberg, 2011), calculating Wald chi‐square statistics for models in *lme* and F‐ratio statistics for models in *lm*. Post hoc Tukey's tests of main effects were carried out using the *glht* function in the package *multcomp* (Hothorn, Bretz, & Westfall, 2008), with main effects averaged over covariates.

We excluded from all analyses four monoculture subplots where no emergence was observed and no individuals were harvested. Furthermore, one *Bromus carinatus* subplot had more than twice the biomass of the next most productive subplot and was removed as an outlier for analyses of biomass and performance in polyculture.

Emergence and density of focal species

To analyze how emergence time varied with origin, herbivore exclusion treatment, and block, a linear mixed‐effects model was built with species nested in origin treated as a random factor. The block effect was nonsignificant (Table S3), and this term was removed from the final model. A second mixed‐effects model was used to analyze how density in monoculture subplots varied with origin, exclusion treatment, and block; species nested in origin was treated as a random factor. Again, there was no significant effect of block (Table S4), and this term was removed from the final model.

*Hypothesis 1*: Earlier‐emerging species are more susceptible to herbivores, and this susceptibility is greatest early in the growing season

To analyze how total biomass in monoculture varied with origin, exclusion treatment, emergence time (and their interactions), as well as plant density and block, a linear mixed‐effects model was built with species nested in origin treated as a random factor. We observed no significant effect of block (Table S5); therefore, this term was removed from the final model. A linear model was used to analyze how total biomass in polyculture subplots varied with exclusion treatment, density, and block; because polyculture subplots contained all species, there were no random effects in this model. We observed no significant effect of block (Table S6); therefore, this term was removed from the final model.

*Hypothesis 2*: In polyculture, the competitive advantage of early‐arriving exotic species is reduced by herbivory, and this effect is greatest early in the growing season

A linear mixed‐effect model was employed to analyze how RPP of focal species varied with origin, exclusion treatment, and emergence time (and their interactions); species nested in origin was treated as a random factor. Because emergence was not monitored in polyculture subplots, mean emergence times of species across all exclusion treatments in monoculture were used; this approach was justified as exclosures did not influence time to emergence for any species (Table S7). Data from all plots in exclusion treatments were pooled to calculate mean individual biomass; therefore, block was not specified in the model.

To confirm that potential differences in mean biomass were driven by herbivory effects rather than differences in density between monoculture and polyculture subplots, a linear mixed‐effects model was constructed to analyze whether mean individual biomass varied with both subplot type (monoculture or polyculture) and density in subplots; species nested within origin was specified as a random factor. Across all subplots, there was no significant effect of subplot type, density, or the interaction between them on mean individual biomass (Table S8). Scatter plots of mean individual biomass in relation to total subplot density in monoculture and polyculture for the 12 focal species are shown in Figure S1.

## 3 Results

### 3.1 Emergence and density of focal species

**Figure 2:**
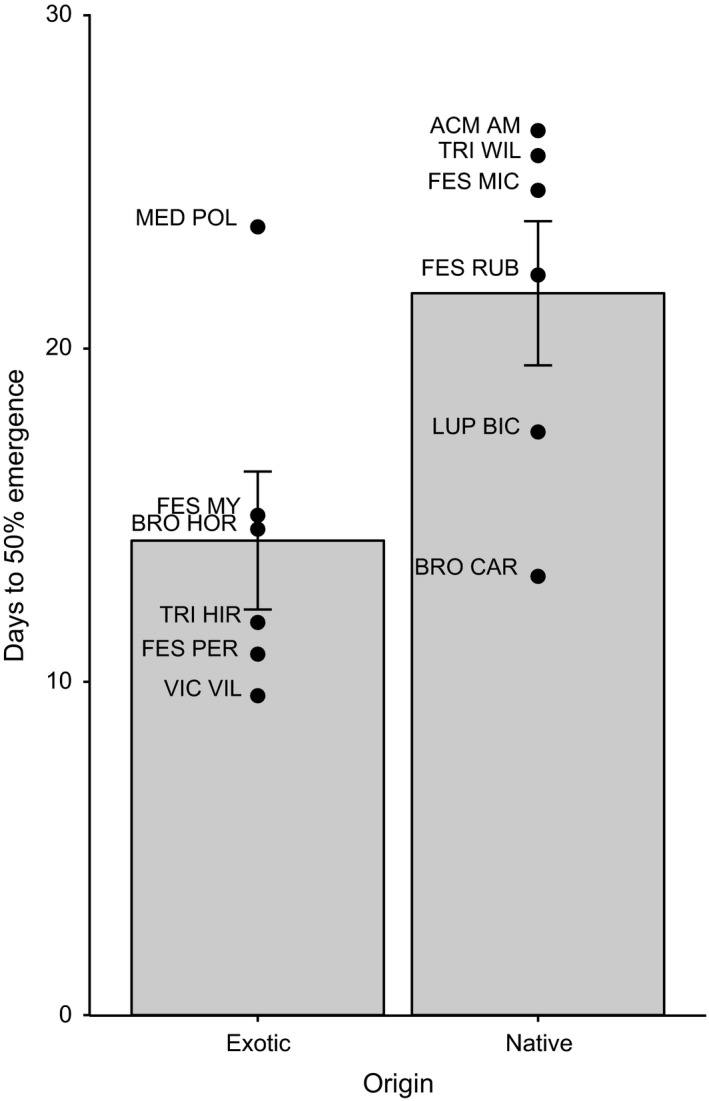
Days to reach 50% emergence for native and exotic focal species. Filled circles represent species mean values. Error bars denote one standard error of the mean where *n* = 6, the number of species within each origin

Consistent with prior work in this study system, exotic species emerged significantly faster than native species (χ^2^_1_ = 4.05, *p* = .044; Table 2; Figure 2). There was no difference in mean emergence time between exclusion treatments (χ^2^_2_ = 4.58, *p* = .10; Table 2) and no significant interaction between origin and exclusion treatment (χ^2^_2_ = 1.61, *p* = .45; Table 2). These results suggest that the following analyses of biomass and RPP were unlikely to be confounded by altered emergence behavior between exclusion treatments. Mean emergence time for species ranged from 9.6 days for the exotic legume *Vicia villosa*, to 26.5 days for the native legume *Acmispon americanus* (Figure 2).

**Table 2:** Analysis‐of‐deviance table derived from linear mixed‐effects model of emergence time in monoculture by origin and exclusion treatment

	Emergence Time		
	*df*	χ^2^	*p*
Origin	1	4.05	.044
Exclusion	2	4.58	.10
Origin × exclusion	2	1.61	.45

Species nested within origin was treated as a random factor.

Overall, we observed mean densities of 13.6 individuals in monoculture and 20.2 individuals in polyculture; however, there was considerable variation among species (Table S2). Exclusion treatment did not significantly affect the density of individuals in monoculture (χ^2^_2_ = 1.57, *p* = .46; Table 3). Native and exotic species did not differ with regard to density (χ^2^_1_ = 1.31, *p* = .25; Table 3), and there was no interaction between origin and exclusion treatment (χ^2^_2_ = 2.36, *p* = .31; Table 3).

**Table 3:** Analysis‐of‐deviance table derived from linear mixed‐effects model of density in monoculture subplots by origin and exclusion treatment

	Density		
	*df*	χ^2^	*p*
Origin	1	1.31	.25
Exclusion	2	1.57	.46
Origin × exclusion	2	2.36	.31

Species nested within origin was treated as a random factor.

### 3.2 Hypothesis 1: Earlier‐emerging species are more susceptible to herbivores, and this susceptibility is greatest early in the growing season

**Figure 3:**
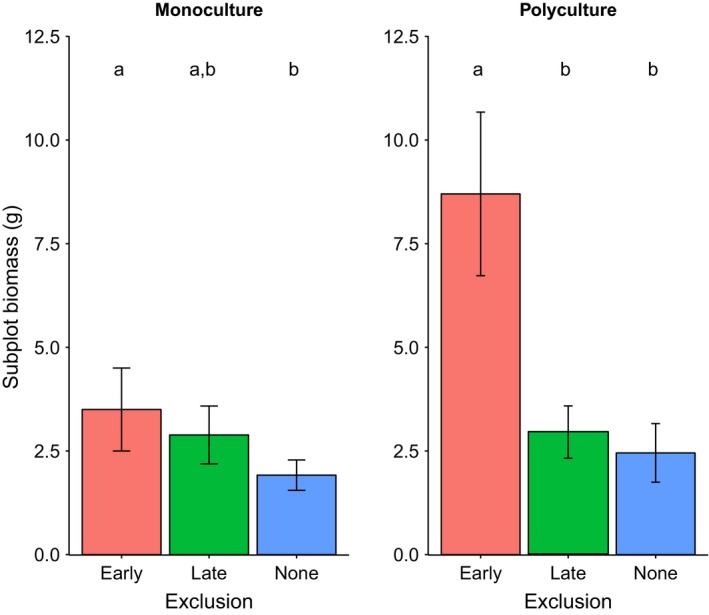
Mean biomass after 70 days in monoculture subplots (left) and polyculture subplots (right). Error bars represent one standard error of the mean: in monoculture *n* = 12, the number of species in each exclusion treatment; in polyculture *n* = 8, the number of replicates within each exclusion treatment. Groups sharing letters do not differ significantly in post hoc Tukey's tests

**Figure 4:**
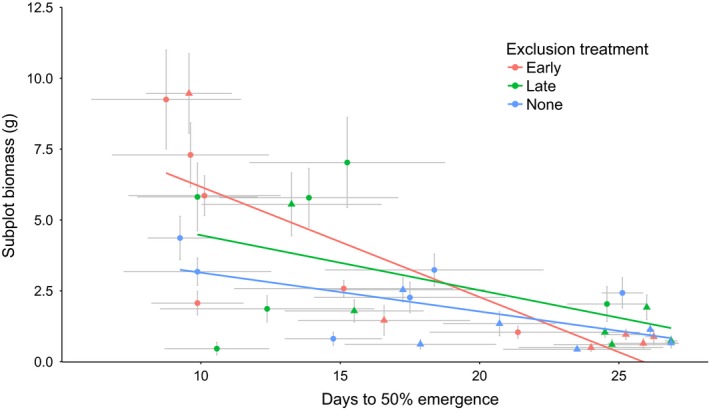
Scatter plot of monoculture subplot biomass in relation to emergence time for species in each exclusion treatment. Exotic species are denoted by circles, and native species are denoted by triangles. Horizontal and vertical gray bars denote one standard error of the mean, where *n* is the number of replicates within each exclusion treatment for each species

Emergence time was a significant predictor of total biomass in monoculture subplots after 70 days, with earlier emergence resulting in greater biomass (χ^2^_1_ = 9.01, *p* = .003; Table 4; Figure 4). Density of plants was a significant predictor of subplot biomass in monoculture (χ^2^_1_ = 22.30, *p *< .001; Table 4), but not in polyculture subplots (*F*_1_ = 1.07, *p* = .31; Table 5). Exclusion treatment was a significant predictor of subplot biomass in both monoculture (χ^2^_2_ = 27.51, *p *< .001; Table 4; Figure 3) and polyculture (*F*_2_ =  8.09, *p* = .003; Table 5; Figure 3). Post hoc Tukey's HSD tests showed that in monoculture, biomass in early exclusion treatments was significantly higher than no exclusion (*p* = .001), while all other pairwise comparisons were nonsignificant (Figure 3). In polyculture, subplot biomass in the early exclusion treatment was significantly higher than both late (*p* = .009) and no exclusion treatments (*p* = .004), with these latter two groups not differing significantly from one another (Figure 3). In monoculture, we observed a significant interaction between emergence time and herbivore exclusion treatment (χ^2^_2_ = 28.63, *p* < .001; Table 4), with earlier emergence associated with greater increases in biomass in early exclusion subplots compared to late and no exclusion subplots (Figure 4). Biomass of native and exotic species in monoculture responded similarly to exclusion treatments, as there was no significant interaction between origin and exclusion treatment (χ^2^_2_ = 2.34, *P* = .31; Table 4).

**Table 4:** Analysis‐of‐deviance table derived from linear mixed‐effects model of subplot biomass in monoculture by origin, exclusion treatment, emergence time, and density

	Biomass		
	*df*	χ^2^	*p*
Origin	1	2.62	.11
Exclusion	2	27.51	<.001
Emergence	1	9.01	.003
Density	1	22.30	<.001
Origin × exclusion	2	2.34	.31
Origin × emergence	1	1.17	.28
Exclusion × emergence	2	28.63	<.001
Origin × exclusion × emergence	2	0.68	.71

Species nested within origin was treated as a random factor.

**Table 5:** Analysis‐of‐variance table derived from linear fixed‐effects model of subplot biomass in polyculture by exclusion treatment and density

	Biomass		
	*df*	*F*	*p*
Exclusion	2	8.09	.003
Density	1	1.07	.31
Residuals	20		

3.3

**Figure 5:**
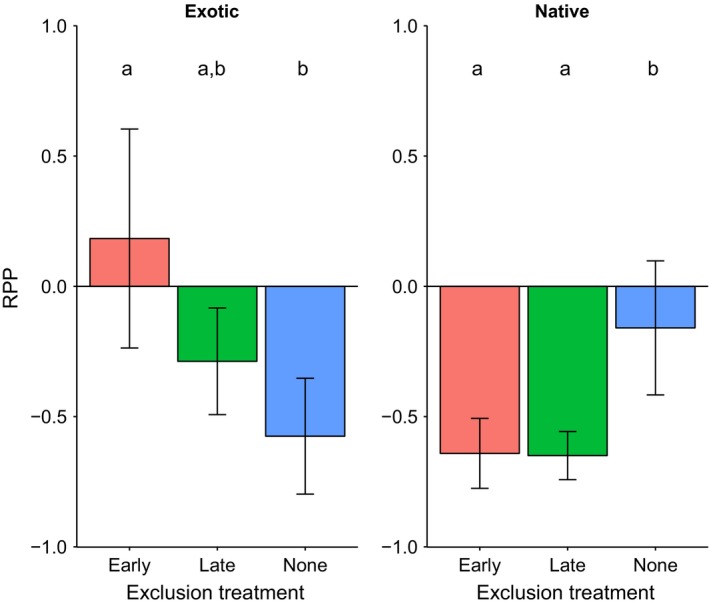
Relative performance in polyculture (RPP) in each exclusion treatment for exotic species (left) and native species (right). Error bars represent one standard error of the mean, where *n* = 6, the number of species in each exclusion treatment. Groups sharing letters do not differ significantly in post hoc Tukey's tests

Hypothesis 2: In polyculture, the competitive advantage of early‐arriving exotic species is reduced by herbivory, and this effect is greatest early in the growing seasonWe observed lower individual biomass in polyculture (relative to monoculture) across all species and exclusion treatments with a mean RPP of −0.355 (±0.106), likely due to the greater density at which individuals were planted in polyculture. We observed no significant effect of emergence time (χ^2^_1_ = 1.76, *p* = .19; Table 6) or exclusion treatment (χ^2^_2_ = 2.11, *p* = .35; Table 6) on RPP, and there was no significant interaction between them (χ^2^_2_ = 1.87, *p* = .39; Table 6). There was no overall difference in RPP between natives and exotics (χ^2^_1_ = 0.008, *p* = .93; Table 6). However, we observed a marginally significant interaction between origin and exclusion treatment (χ^2^_2_ = 5.81, *p* = .055; Table 6; Figure 5). Post hoc Tukey's HSD tests showed that for exotic species, RPP was significantly higher in the early exclusion treatment than in the no exclusion treatment (*p* = .013), while all other pairwise comparisons were nonsignificant (Figure 5). For native species, RPP was significantly higher in the no exclusion treatment than in the early exclusion (*p* = .037) and late exclusion treatments (*p* = .033) (Figure 5).

**Table 6:** Analysis‐of‐deviance table derived from linear mixed‐effects model of RPP by origin, exclusion treatment, and emergence time

	RPP		
	*df*	χ^2^	*p*
Origin	1	0.008	.93
Exclusion	2	2.11	.35
Emergence	1	1.76	.19
Origin × exclusion	2	5.81	.055
Origin × emergence	1	0.43	.51
Exclusion × emergence	2	1.87	.39
Origin × exclusion × emergence	2	0.20	.91

Species nested within origin was treated as a random factor.

## 4 Discussion

### 4.1 Emergence and density of focal species

Exotic species emerged faster than native species, consistent with past observations in California (Abraham et al., 2009; Deering & Young, 2006; Reynolds et al., 2001; Wainwright & Cleland, 2013; Wainwright et al., 2012). Although evidence is accumulating that emergence time is phylogenetically conserved (Norden et al., 2009; Xu, Li, Zhang, Liu, & Du, 2014), we observed significant differences between natives and exotics even when the species pool was taxonomically balanced at the family level.

The density of individuals in monoculture did not differ significantly across exclusion treatments. The differences in biomass between exclusion treatments are largely the result of differences in the size of plants harvested, rather than differences in the number of individuals remaining after 70 days.

### 4.2 Hypothesis 1: Earlier‐emerging species are more susceptible to herbivores, and this susceptibility is greatest early in the growing season

As expected, earlier emergence resulted in greater biomass in monoculture subplots. This may be because such species have a longer time to accrue biomass, but could also reflect higher growth rates of species that emerge earlier. The higher overall biomass observed when herbivores were excluded early in the growing season (Figure 3) confirms that herbivory at this stage has disproportionately large effects on plant growth; this result is striking given that actively growing plants in the late exclusion treatment were effectively protected from herbivory for longer than plants in the early exclusion treatment (because the first 35 days of exclusion started with the very first emerging individuals, and most individuals emerged after this date). The significant interaction between emergence time and exclusion treatment (Figure 4) shows that this higher overall impact of herbivory in the early season is driven largely by the increased susceptibility of earlier‐emerging species which tended to have greater biomass. This result therefore lends support to our first hypothesis of a trade‐off between emergence time and susceptibility to herbivory that is most prominent in the early growing season.

The greater impact of herbivory in the early growing season is likely due to greater herbivore preference for younger plants and lower ability to tolerate damage in earlier ontogenetic stages. Herbivores often prefer tissue of younger plants because they are less strongly defended and of greater nutritional quality (Quintero et al., 2014). Furthermore, younger plants may have limited capacity to regrow in response to herbivory (Boege & Marquis, 2005; Strauss & Agrawal, 1999). Simulated herbivory in European grassland species has been shown to be most detrimental to growth when occurring earliest in ontogeny, with lower biomass persisting for longer than the duration of this experiment (Hanley & Fegan, 2007). It must be noted that tolerance to herbivory also reflects the ability to reproduce following damage, and this is expected to be low if damage occurs shortly before reproduction (Strauss & Agrawal, 1999). As plants were harvested before reaching reproductive maturity, the fitness consequences of the timing of herbivore exposure were beyond the scope of this experiment.

As there was no significant difference in the density of individuals between exclusion treatments, the impacts of herbivory in this experiment were largely mediated by reductions in plant size rather than mortality. Although herbivory is often fatal for plants in early ontogenetic stages, this result is less surprising given that species which emerged earlier and achieved greater biomass were disproportionately impacted, and these would be expected to better tolerate herbivore damage (Boege & Marquis, 2005; Strauss & Agrawal, 1999).

We hypothesized that this increased susceptibility of early‐emerging species could result from greater apparency to herbivores. However, the association between larger size and greater susceptibility observed in this experiment could also result from a trade‐off between growth and defense. With this experimental design, we are unable to conclude that this pattern was observed solely due to differences in apparency. A study in northern California by Cushman et al. (2011) found that herbivory by native black‐tailed jackrabbits on the exotic grass *Ehrharta calycina* was lower when plants were growing in association with established native and exotic perennials which reduced their apparency. Therefore, apparency is likely an important mechanism underlying the trade‐off between early emergence and increased susceptibility to generalist mammalian herbivores observed in this experiment. It must also be noted that herbivore feeding behavior in this experimental context (where there is a choice between treatments which are separated spatially) may not reflect behavior in a more natural, nonchoice situation such as a homogenous grassland.

As all plants were harvested simultaneously after 70 days (regardless of emergence time), the increased susceptibility of earlier‐emerging species to early‐season herbivory might simply be a function of the longer time that they were exposed to herbivores (approximately 17 days between the earliest and latest‐emerging species; Figure 2). However, as discussed above, herbivore impact on biomass was not predicted by the duration of exposure to herbivores: Late‐emerging species showed little response to exclusion treatments that differed in herbivore exposure time by 35 days or more (Figure 4). Another potential driver of the increased susceptibility associated with earlier emergence is that exotic species, which on average emerged earlier, may be less resistant to evolutionarily novel native herbivores when encountered, perhaps because of a lack of coevolved defenses (Colautti et al., 2004; Parker et al., 2006). However, we observed no significant interaction between exclusion treatment and origin, showing that origin per se had little effect on susceptibility in monoculture.

### 4.3 Hypothesis 2*:* In polyculture, the competitive advantage of early‐arriving exotic species is reduced by herbivory, and this effect is greatest early in the growing season

We found considerable support for our second hypothesis. Exotic species, which emerged faster, had significantly higher RPP when herbivores were excluded early compared to the no exclusion treatment (Figure 5). On the other hand, later‐emerging natives had significantly higher RPP when herbivores were not excluded throughout the experiment (Figure 5). This highlights the importance of accounting for herbivory (or lack thereof) when interpreting results from priority effects studies.

These patterns of RPP between exclusion treatments are likely observed because differences in emergence time tend to be larger between heterospecific competitors than conspecifics, which has several consequences. Firstly, individuals of early‐emerging exotic species in polyculture can preempt resources to a greater extent because they are competing with heterospecifics that are more likely to emerge later. This could lead to improved performance of early‐emerging exotic species in polyculture, consistent with priority effects mechanisms (Fukami, 2015). On the other hand, later‐emerging native species may display suppressed growth in polyculture because they are competing with heterospecifics that are more likely to emerge earlier. Secondly, the increased differences in emergence time between individuals in polyculture may also enhance herbivory effects. The greater size of exotic species that emerge earlier than heterospecific competitors in polyculture may mean they are relatively more apparent and accessible than when growing with conspecifics in monoculture, a form of associational susceptibility (Underwood et al., 2014). Smaller, later‐emerging native species however would be relatively less apparent and accessible when growing with larger heterospecific competitors in polyculture than with similar sized conspecifics in monoculture; therefore, growth in polyculture may provide associational resistance (Underwood et al., 2014). Furthermore, native species can benefit from competitive release if early‐active exotic species are preferentially consumed (Beck, Hernández, Pasari, & Zavaleta, 2015).

The greater RPP of native species observed in the no exclusion treatment suggests that in this system mammalian herbivores may play an important role in maintaining the performance of late‐emerging natives in the face of competitive advantages gained by early‐emerging exotics. How these changes in performance translate to population‐level effects on the abundance of native and exotic species is beyond the scope of this experiment, as abundance is ultimately determined by multiple demographic processes such as recruitment and mortality (Crawley, 2007). However, field experiments have shown that in Mediterranean ecosystems, herbivores can indeed reduce the abundance of early‐active exotic species or functional groups and increase the abundance of later‐active species. For example, in California grasslands, decreases in the abundance of early‐active exotic annual grasses as a result of exposure to herbivores are linked to increased abundance of forb species that tend to emerge slowly in response to seasonal rains (Peters, 2007; Skaer, Graydon, & Cushman, 2013). Grazing in this system has also been shown to increase the cover and diversity of native forbs as well as decreasing year‐to‐year variability of these metrics (Beck et al., 2015).

Our results indicate that emergence time represents a trade‐off between competitive ability and herbivore susceptibility and that native and exotic species differ in their strategies. Fast emergence of exotics may allow them to benefit from seasonal priority effects (Wainwright et al., 2012), but at the cost of increased susceptibility to generalist herbivores. Previous studies have shown that exotic species in Mediterranean ecosystems are better able to tolerate herbivore damage than natives, perhaps due to their longer evolutionary histories of intensive grazing (Hille Ris Lambers, Yelenik, Colman, & Levine, 2010; Holmgren, 2002). This may allow them to maintain a strategy of early emergence relative to the native community, despite their increased susceptibility to mammalian herbivores.

### 4.4 Shade effects of herbivore exclosures

Although species emergence times were not affected by exclusion treatment, a caveat of this experiment is that exclosures may also have altered biomass and RPP by reducing light availability (30% reduction in PAR). It has been shown in grassland systems that community biomass can increase under conditions of moderate shade (50%), with many species responding positively as result of stress reduction (Semchenko, Lepik, Götzenberger, & Zobel, 2012). Although the shading in this experiment is considerably lower, it is still possible that this may have affected biomass results over and above the effect of herbivore exclusion. If the partial shade provided by exclosures was a major factor promoting growth, then early‐emerging species would likely benefit more from early exclusion than later‐emerging species, as they have a relatively longer period of growth under shade. However, biomass would also be expected to be greatest in the late exclusion treatment where actively growing plants were effectively covered for longest. Similarly, if the contrasting RPP responses of native and exotics to herbivore exclusion treatments were driven by differential capacity to tolerate shade, then the biggest differences in RPP would be expected between late and no exclusion treatments where the effective duration of shade experienced by actively growing plants was longest and shortest, respectively. Although we cannot rule out the possibility that shade influenced differences in biomass and RPP between exclusion treatments, there is little evidence suggesting that these effects, rather than herbivory, were driving observed patterns.

### 4.5 Management implications

Herbivores can have contrasting effects on exotic plant invasions in Mediterranean ecosystems. For example, continuous, heavy grazing over many years is thought to have contributed to the invasion of Chile, southwestern Australia, and California by exotic herbaceous species, many of which were likely preadapted to tolerate this chronic disturbance (Hille Ris Lambers et al., 2010; Hobbs, 2001; Holmgren, 2002). Despite having the potential to facilitate invasions, grazing is often suggested as an effective conservation and restoration tool to minimize exotic impacts in such regions (e.g., Beck et al., 2015). This is because it is becoming increasingly recognized that phenological differences between native and exotic species can be exploited to enhance the success of management and restoration efforts (Marushia, Cadotte, & Holt, 2010; Wolkovich & Cleland, 2011), and therefore prescription of grazing treatments during appropriate phenological windows can lead to more favorable outcomes for native species (Rinella & Hileman, 2009). For example, short‐duration grazing while exotic species are flowering is an effective restoration tool that acts by reducing their seed output (Menke, 1992). Our results suggest that, in areas characterized by rapid exotic emergence, grazing during the earliest period of the growing season is likely to be another effective management strategy for improving native performance relative to exotic competitors. This is consistent with findings of studies in California showing that grazing in the winter/spring (early season) leads to greater decreases in the abundance of early‐active exotics (Skaer et al., 2013), whereas grazing applied in the spring/summer (late season), at which point many exotic species are no longer active, leads to greater negative impacts on native abundance (Hille Ris Lambers et al., 2010). However, early‐season grazing is unlikely to be effective where exotic species do not benefit from priority effects (Funk, Hoffacker, & Matzek, 2015).

## 5 Conclusions

This study took place in the Mediterranean‐type climate region of southern California where seasonal growth is initiated following the arrival of winter rains. However, the factors controlling the onset of the growing season vary within and across biomes, leading to differences in growing season length and speed of seasonal transitions (Pau et al., 2011). We expect that the trade‐off between emergence time and herbivore susceptibility observed in this experiment will be present in all systems where growth occurs within distinct growing seasons, regardless of the factors determining their onset. In regions where growth is year‐round, we would not expect to see a relationship between emergence time and herbivore susceptibility. What is less clear is how the length of growing seasons and the speed of seasonal transitions determine the magnitude of this trade‐off. For example, slower seasonal transitions might result in larger differences in emergence time between early‐and late‐arriving species (Pau et al., 2011), and this may enhance both the competitive advantage and increased herbivore susceptibility associated with early arrival.

The proposed mechanism by which early‐active species are more susceptible to herbivores (increased apparency) might conceivably also result in increased susceptibility for species that have extended late‐season phenology. While this was beyond the scope of this short‐term experiment, future work could explore whether an analogous trade‐off exists between extended phenology and herbivore susceptibility. Such understanding would be particularly valuable given that extended phenology has been identified as a key trait in certain Mediterranean invaders such as *Centaurea solstitialis* (Roché & Thill, 2001), and invaders in other systems including deciduous forests (Fridley, 2012).

This study demonstrates that in systems defined by seasonal periods of growth, emergence time is a key trait that may predict species’ susceptibility to generalist herbivores. Because herbivore defense and phenology are both associated with many other correlated plant functional traits (Wolkovich & Cleland, 2014), this trade‐off is likely an important driver of the evolution of life‐history variation in seasonally defined communities. In addition to aiding our fundamental understanding of how trade‐offs predict variation in species abundances and species coexistence in diverse communities, an understanding of trade‐offs may improve the efficacy of management efforts that exploit phenological differences between native and exotic species.

## Data accessibility

Data are intended for archive in the Knowledge Network for Biocomplexity (KNB) https://knb.ecoinformatics.org/.

## Conflict of Interest

None declared.

## References

[ece32610-bib-0001] Abraham, J. K. , Corbin, J. D. , & D'Antonio, C. M. (2009). California native and exotic perennial grasses differ in their response to soil nitrogen, exotic annual grass density, and order of emergence. Plant Ecology, 201, 445–456.

[ece32610-bib-0002] Anderson, J. T. , Inouye, D. W. , McKinney, A. M. , Colautti, R. I. , & Mitchell‐Olds, T. (2012). Phenotypic plasticity and adaptive evolution contribute to advancing flowering phenology in response to climate change. Proceedings of the Royal Society B, 279, 3843–3852. 22787021 10.1098/rspb.2012.1051 PMC3415914

[ece32610-bib-0003] Augspurger, C. (2013). Reconstructing patterns of temperature, phenology, and frost damage over 124 years: Spring damage risk is increasing. Ecology, 94, 41–50. 23600239 10.1890/12-0200.1

[ece32610-bib-0004] Bartolome, J. W. (1979). Germination and seedling establishment in California annual grassland. Journal of Ecology, 67, 273–281.

[ece32610-bib-0005] Barton, K. E. , & Hanley, M. E. (2013). Seedling‐herbivore interactions: Insights into plant defence and regeneration patterns. Annals of Botany, 112, 643–650. 23925939 10.1093/aob/mct139 PMC3736773

[ece32610-bib-0006] Beck, J. J. , Hernández, D. L. , Pasari, J. R. , & Zavaleta, E. S. (2015). Grazing maintains native plant diversity and promotes community stability in an annual grassland. Ecological Applications, 25, 1259–1270. 26485954 10.1890/14-1093.1

[ece32610-bib-0007] Boege, K. , & Marquis, R. J. (2005). Facing herbivory as you grow up: The ontogeny of resistance in plants. Trends in Ecology and Evolution, 20, 441–448. 16701415 10.1016/j.tree.2005.05.001

[ece32610-bib-0008] Burt‐Smith, G. S. , Grime, J. P. , & Tilman, D. (2003). Seedling resistance to herbivory as a predictor of relative abundance in a synthesised prairie community. Oikos, 101, 345–353.

[ece32610-bib-0009] Cleland, E. E. , Esch, E. , & McKinney, J. (2015). Priority effects vary with species identity and origin in an experiment varying the timing of seed arrival. Oikos, 124, 33–40.

[ece32610-bib-0010] Cleland, E. E. , Funk, J. L. , & Allen, E. B. (2016). Coastal Sage Scrub In Mooney H. A., & Zavaleta E. S. (Eds.), Ecosystems of California (pp. 429–448). Berkeley, California, USA: University of California Press.

[ece32610-bib-0011] Cleland, E. E. , Peters, H. A. , Mooney, H. A. , & Field, C. B. (2006). Gastropod herbivory in response to elevated CO_2_ and N addition impacts plant community composition. Ecology, 87, 686–694. 16602298 10.1890/05-0529

[ece32610-bib-0012] Colautti, R. I. , Ricciardi, A. , Grigorovich, I. A. , & MacIsaac, H. J. (2004). Is invasion success explained by the enemy release hypothesis? Ecology Letters, 7, 721–733.

[ece32610-bib-0013] Crawley, M. J. (2007). Plant population dynamics In May R. M., & McLean A. (Eds.), Theoretical ecology: Principles and applications (pp. 62–83). Oxford: Oxford University Press.

[ece32610-bib-0014] Cushman, J. H. , Lortie, C. J. , & Christian, C. E. (2011). Native herbivores and plant facilitation mediate the performance and distribution of an invasive exotic grass. Journal of Ecology, 99, 524–531.

[ece32610-bib-0015] Deering, R. H. , & Young, T. P. (2006). Germination speeds of exotic annual and native perennial grasses in California and the potential benefits of seed priming for grassland restoration. Grasslands, 16, 14–16.

[ece32610-bib-0016] Fenner, M. , & Thompson, K. (2005). The ecology of seeds. Cambridge: Cambridge University Press.

[ece32610-bib-0017] Fox, J. , & Weisberg, S. (2011). An R companion to applied regression, 2nd ed. Thousand Oaks, California, USA: Sage.

[ece32610-bib-0018] Fridley, J. D. (2012). Extended leaf phenology and the autumn niche in deciduous forest invasions. Nature, 485, 359–362. 22535249 10.1038/nature11056

[ece32610-bib-0019] Fukami, T. (2015). Historical contingency in community assembly: Integrating niches, species pools, and priority effects. Annual Review of Ecology, Evolution, and Systematics, 46, 1–23.

[ece32610-bib-0020] Funk, J. L. , Hoffacker, M. K. , & Matzek, V. (2015). Summer irrigation, grazing and seed addition differentially influence community composition in an invaded serpentine grassland. Restoration Ecology, 23, 122–130.

[ece32610-bib-0021] Godoy, O. , Valladares, F. , & Castro‐Díez, P. (2011). Multispecies comparison reveals that invasive and native plants differ in their traits but not in their plasticity. Functional Ecology, 25, 1248–1259.

[ece32610-bib-0022] Grman, E. , & Suding, K. N. (2010). Within‐year soil legacies contribute to strong priority effects of exotics on native California grassland communities. Restoration Ecology, 18, 664–670.

[ece32610-bib-0023] Hanley, M. E. (1998). Seedling herbivory, community composition and plant life history traits. Perspectives in Plant Ecology, Evolution and Systematics, 1, 191–205.

[ece32610-bib-0024] Hanley, M. E. , & Fegan, E. L. (2007). Timing of cotyledon damage affects growth and flowering in mature plants. Plant, Cell & Environment, 30, 812–819. 10.1111/j.1365-3040.2007.01671.x 17547653

[ece32610-bib-0025] Hanley, M. E. , & Sykes, R. J. (2009). Impacts of seedling herbivory on plant competition and implications for species coexistence. Annals of Botany, 103, 1347–1353. 19351683 10.1093/aob/mcp081 PMC2685311

[ece32610-bib-0026] Heady, H. F. (1958). Vegetational changes in the California annual type. Ecology, 39, 402–416.

[ece32610-bib-0027] Hille Ris Lambers, J. , Yelenik, S. G. , Colman, B. P. , & Levine, J. M. (2010). California annual grass invaders: The drivers or passengers of change? Journal of Ecology, 98, 1147–1156. 20852668 10.1111/j.1365-2745.2010.01706.x PMC2936119

[ece32610-bib-0028] Hobbs, R. J. (2001). Synergisms among habitat fragmentation, livestock grazing, and biotic invasions in southwestern Australia. Conservation Biology, 15, 1522–1528.

[ece32610-bib-0029] Holmgren, M. (2002). Exotic herbivores as drivers of plant invasion and switch to ecosystem alternative states. Biological Invasions, 4, 25–33.

[ece32610-bib-0030] Hothorn, T. , Bretz, F. , & Westfall, P. (2008). Simultaneous inference in general parametric models. Biometrical Journal, 50, 346–363. 18481363 10.1002/bimj.200810425

[ece32610-bib-0031] Hulme, P. E. (1994). Seedling herbivory in grassland: Relative impact of vertebrate and invertebrate herbivores. Journal of Ecology, 82, 873–880.

[ece32610-bib-0032] Keane, R. M. , & Crawley, M. J. (2002). Exotic plant invasions and the enemy release hypothesis. Trends in Ecology and Evolution, 17, 164–170.

[ece32610-bib-0033] Kempel, A. , Razanajatovo, M. , Stein, C. , Unsicker, S. B. , Auge, H. , Weisser, W. W. … Prati, D. (2015). Herbivore preference drives plant community composition. Ecology, 96, 2923–2934. 27070012 10.1890/14-2125.1

[ece32610-bib-0034] van Kleunen, M. , Weber, E. , & Fischer, M. (2010). A meta‐analysis of trait differences between invasive and non‐invasive plant species. Ecology Letters, 13, 235–245. 20002494 10.1111/j.1461-0248.2009.01418.x

[ece32610-bib-0035] Lambrinos, J. G. (2006). Spatially variable propagule pressure and herbivory influence invasion of chaparral shrubland by an exotic grass. Oecologia, 147, 327–334. 16189663 10.1007/s00442-005-0259-1

[ece32610-bib-0036] Leishman, M. R. , Haslehurst, T. , Ares, A. , & Baruch, Z. (2007). Leaf trait relationships of native and invasive plants: community‐ and global‐ scale comparisons. New Phytologist, 176, 635–643. 17822409 10.1111/j.1469-8137.2007.02189.x

[ece32610-bib-0037] Loreau, M. (1998). Separating sampling and other effects in biodiversity experiments. Oikos, 82, 600–602.

[ece32610-bib-0038] Marushia, R. G. , Cadotte, M. W. , & Holt, J. S. (2010). Phenology as a basis for management of exotic annual plants in desert invasions. Journal of Applied Ecology, 47, 1290–1299.

[ece32610-bib-0039] Menke, J. W. (1992). Grazing and fire management for native perennial grass restoration in California grasslands. Fremontia, 20, 22–25.

[ece32610-bib-0040] Norden, N. , Daws, M. I. , Antoine, C. , Gonzalez, M. A. , Garwood, N. C. , & Chave, J. (2009). The relationship between seed mass and mean time to germination for 1037 tree species across five tropical forests. Functional Ecology, 23, 203–210.

[ece32610-bib-0041] Parker, J. D. , Burkepile, D. E. , & Hay, M. E. (2006). Opposing effects of native and exotic herbivores on plant invasions. Science, 311, 1459–1461. 16527979 10.1126/science.1121407

[ece32610-bib-0042] Pau, S. , Wolkovich, E. M. , Cook, B. I. , Jonathan Davies, T. , Kraft, N. J. B. , Bolmgren, K. , … Cleland, E. E. (2011). Predicting phenology by integrating ecology, evolution and climate science. Global Change Biology, 17, 3633–3643.

[ece32610-bib-0043] Pérez‐Fernández, M. A. , Lamont, B. B. , Marwick, A. L. , & Lamont, W. G. (2000). Germination of seven exotic weeds and seven native species in south‐western Australia under steady and fluctuating water supply. Acta Oecologica, 21, 323–336.

[ece32610-bib-0044] Peters, H. A. (2007). The significance of small herbivores in structuring annual grassland. Journal of Vegetation Science, 18, 175–182.

[ece32610-bib-0045] Pinheiro, J. , Bates, D. , DebRoy, S. , Sarkar, D. & R Core Team (2015). nlme: linear and nonlinear mixed effects models. R package version 3.1‐121.

[ece32610-bib-0046] Quintero, C. , Lampert, E. C. , & Bowers, M. D. (2014). Time is of the essence: direct and indirect effects of plant ontogenetic trajectories on higher trophic levels. Ecology, 95, 2589–2602.

[ece32610-bib-0047] R Core Team . (2015). R: A language and environment for statistical computing. Vienna, Austria: R Foundation for Statistical Computing.

[ece32610-bib-0048] Reynolds, S. A. , Corbin, J. D. , & D'Antonio, C. M. (2001). The effects of litter and temperature on the germination of native and exotic grasses in a coastal California grassland. Madroño, 48, 230–235.

[ece32610-bib-0049] Rice, K. J. (1987). Interaction of disturbance patch size and herbivory in *Erodium* colonization. Ecology, 68, 1113–1115.

[ece32610-bib-0050] Rinella, M. J. , & Hileman, B. J. (2009). Efficacy of prescribed grazing depends on timing intensity and frequency. Journal of Applied Ecology, 46, 796–803.

[ece32610-bib-0051] Roché, C. T. , & Thill, D. C. (2001). Biology of common crupina and yellow starthistle, two Mediterranean winter annual invaders in western North America. Weed Science, 49, 439–447.

[ece32610-bib-0052] Sala, O. E. , Chapin, F. S. III , Armesto, J. J. , Berlow, R. , Bloomfield, J. , Dirzo, R. , … Wall, D. H. (2000). Global biodiversity scenarios for the year 2100. Science, 287, 1770–1774. 10710299 10.1126/science.287.5459.1770

[ece32610-bib-0053] Semchenko, M. , Lepik, M. , Götzenberger, L. , & Zobel, K. (2012). Positive effect of shade on plant growth: Amelioration of stress or active regulation of growth rate? Journal of Ecology, 100, 459–466.

[ece32610-bib-0054] Skaer, M. J. , Graydon, D. J. , & Cushman, J. H. (2013). Community‐level consequences of cattle grazing for an invaded grassland: Variable responses of native and exotic vegetation. Journal of Vegetation Science, 24, 332–343.

[ece32610-bib-0055] Strauss, S. , & Agrawal, A. (1999). The ecology and evolution of plant tolerance to herbivory. Trends in Ecology and Evolution, 14, 179–185. 10322530 10.1016/s0169-5347(98)01576-6

[ece32610-bib-0056] Sullivan, A. T. , & Howe, H. F. (2009). Prairie forb response to timing of vole herbivory. Ecology, 90, 1346–1355. 19537554 10.1890/08-0629.1

[ece32610-bib-0057] Underwood, N. , Inouye, B. D. , & Hambäck, P. A. (2014). A conceptual framework for associational effects: When do neighbors matter and how would we know? The Quarterly Review of Biology, 89, 1–19. 24672901 10.1086/674991

[ece32610-bib-0058] Underwood, E. C. , Viers, J. H. , Klausmeyer, K. R. , Cox, R. L. , & Shaw, M. R. (2009). Threats and biodiversity in the Mediterranean biome. Diversity and Distributions, 15, 188–197.

[ece32610-bib-0059] Vaughn, K. J. , & Young, T. P. (2015). Short‐term priority over exotic annuals increases the initial density and longer‐term cover of native perennial grasses. Ecological Applications, 25, 791–799. 26214923 10.1890/14-0922.1

[ece32610-bib-0060] Vilà, M. , & D'Antonio, C. M. (1998). Hybrid vigor for clonal growth in *Carpobrotus* (Aizoaceae) in coastal California. Ecological Applications, 8, 1196–1205.

[ece32610-bib-0061] Wainwright, C. E. , & Cleland, E. E. (2013). Exotic species display greater germination plasticity and higher germination rates than native species across multiple cues. Biological Invasions, 15, 2253–2264.

[ece32610-bib-0062] Wainwright, C. E. , Wolkovich, E. M. , & Cleland, E. E. (2012). Seasonal priority effects: Implications for invasion and restoration in a semi‐arid system. Journal of Applied Ecology, 49, 234–241.

[ece32610-bib-0063] Wolkovich, E. M. , & Cleland, E. E. (2011). The phenology of plant invasions: A community ecology perspective. Frontiers in Ecology and the Environment, 9, 287–294.

[ece32610-bib-0064] Wolkovich, E. M. , & Cleland, E. E. (2014). Phenological niches and the future of invaded ecosystems with climate change. AoB PLANTS, 6, plu013; doi:10.1093/aobpla/plu013 10.1093/aobpla/plu013 PMC4025191 24876295

[ece32610-bib-0065] Xu, J. , Li, W. , Zhang, C. , Liu, W. , & Du, G. (2014). Variation in seed germination of 134 common species on the eastern Tibetan Plateau: Phylogenetic, life history and environmental correlates. PLoS ONE, 9(6), e98601. doi:10.1371/journal.pone.0098601. 24893308 10.1371/journal.pone.0098601 PMC4043731

