# Supplementary material for: Trade‐off between early emergence and herbivore susceptibility mediates exotic success in an experimental California plant community
Source: Ecol Evol. 2016 Nov 30;6(24):8942–53. doi: 10.1002/ece3.2610 (PMC5192797; doi:10.1002/ece3.2610)
Supplement: Supplementary file 1 [file ECE3-6-8942-s001.docx]

Supporting information for: “Trade-off between early emergence and herbivore susceptibility mediates exotic success in an experimental California plant community”.

Table S1. Photosynthetically active radiation (PAR) inside and outside herbivore exclosures.

| Measurement Date | Outside Exclosure (μmol m^-2^s^-1^) | Inside Exclosure  (μmol m^-2^s^-1^) | Exclosure PAR Reduction (%) |
| --- | --- | --- | --- |
| 02/18/2015 | 1030 | 710 | 31.07 |
| 04/03/2015 | 1220 | 828 | 32.13 |

Table S2. Seeds planted, mean emergents after 35 days and mean number of individuals harvested after 70 days for 12 focal species across all monoculture and polyculture subplots.

| Scientific name | Monoculture | | | | Polyculture | |
| --- | --- | --- | --- | --- | --- | --- |
|  | Seeds Planted | Total Emergents at 35 Days | Percent Emergence at 35 days | Individuals Harvested at 70 days (Density) | Seeds Planted | Individuals Harvested at 70 Days |
| *Acmispon americanus* | 41 | 11.5 | 28.1 | 10.3 | 6 | 1.6 |
| *Bromus carinatus* | 26 | 17.3 | 66.5 | 17.3 | 4 | 1.6 |
| *Bromus hordeaceus* | 16 | 12.0 | 75.3 | 12.3 | 2 | 2.2 |
| *Festuca microstachys* | 20 | 11.8 | 59.0 | 10.8 | 3 | 2.2 |
| *Festuca myuros* | 18 | 11.7 | 64.8 | 12.2 | 3 | 1.5 |
| *Festuca perennis* | 16 | 12.5 | 77.9 | 11.7 | 2 | 1.1 |
| *Festuca rubra* | 32 | 15.3 | 47.8 | 15.6 | 5 | 1.5 |
| *Lupinus bicolor* | 41 | 7.4 | 18.1 | 6.5 | 6 | 0.6 |
| *Medicago polymorpha* | 35 | 12.1 | 34.6 | 8.9 | 5 | 0.8 |
| *Trifolium hirtum* | 32 | 7.9 | 24.6 | 5.0 | 5 | 1.0 |
| *Trifolium willdenovi* | 233 | 40.1 | 17.2 | 37.7 | 33 | 4.4 |
| *Vicia villosa* | 21 | 13.6 | 64.7 | 13.0 | 3 | 1.9 |

Table S3. Analysis-of-deviance table derived from linear mixed-effects model of emergence time in monoculture by origin and exclusion treatment, with block included as a fixed effect. Species nested within origin was treated as a random factor. Block was non-significant and therefore removed from the final model.

|  | Emergence Time |  |  |
| --- | --- | --- | --- |
|  | Df | χ^2^ | *P* |
| Origin | 1 | 4.00 | 0.046 |
| Exclusion | 2 | 4.59 | 0.10 |
| Block | 7 | 8.67 | 0.28 |
| Origin*Exclusion | 2 | 1.66 | 0.44 |

Table S4. Analysis-of-deviance table derived from linear mixed-effects model of density in monoculture by origin and exclusion treatment, with block included as a fixed effect. Species nested within origin was treated as a random factor. Block was non-significant and therefore removed from the final model.

|  | Density |  |  |
| --- | --- | --- | --- |
|  | Df | χ^2^ | *P* |
| Origin | 1 | 1.31 | 0.25 |
| Exclusion | 2 | 1.50 | 0.47 |
| Block | 7 | 5.98 | 0.54 |
| Origin*Exclusion | 2 | 2.39 | 0.30 |

Table S5. Analysis-of-deviance table derived from linear mixed-effects model of subplot biomass in monoculture by origin, exclusion treatment, emergence time and density, with block included as a fixed effect. Species nested within origin was treated as a random factor. Block was non-significant and therefore removed from the final model.

|  | Biomass |  |  |
| --- | --- | --- | --- |
|  | Df | χ^2^ | *P* |
| Origin | 1 | 2.57 | 0.11 |
| Exclusion | 2 | 27.54 | <0.001 |
| Emergence | 1 | 10.73 | 0.001 |
| Density | 1 | 23.28 | <0.001 |
| Block | 7 | 10.96 | 0.14 |
| Origin*Exclusion | 2 | 2.90 | 0.24 |
| Origin*Emergence | 1 | 0.67 | 0.41 |
| Exclusion*Emergence | 2 | 30.29 | <0.001 |
| Origin*Exclusion*Emergence | 2 | 0.57 | 0.75 |

Table S6. Analysis-of-variance table derived from linear fixed-effects model of subplot biomass in polyculture by exclusion treatment and density, with block included as a fixed effect. Block was non-significant and therefore removed from the final model.

|  | Biomass | |  |  |
| --- | --- | --- | --- | --- |
|  | Df | F | | *P* |
| Exclusion | 2 | 12.30 | | 0.001 |
| Density | 1 | 2.12 | | 0.17 |
| Block | 7 | 2.39 | | 0.083 |
| Residuals | 13 |  | |  |

Table S7. Analysis-of-variance table derived from linear fixed-effects model of emergence time in monoculture by exclusion treatment and species.

|  | Emergence Time | |  |  |
| --- | --- | --- | --- | --- |
|  | Df | F | | *P* |
| Exclusion | 2 | 2.31 | | 0.10 |
| Species | 11 | 20.46 | | <0.001 |
| Exclusion*Species | 22 | 1.10 | | 0.35 |
| Residuals | 248 |  | |  |

Table S8. Analysis-of-deviance table derived from linear mixed-effects model of individual biomass by subplot type (monoculture or polyculture) and density. Species nested within origin was treated as a random factor.

|  | Individual Biomass | |  |  |
| --- | --- | --- | --- | --- |
|  | Df | χ^2^ | | *P* |
| Subplot | 1 | 0.15 | | 0.70 |
| Density | 1 | 2.13 | | 0.14 |
| Subplot*Density | 1 | 0.059 | | 0.81 |


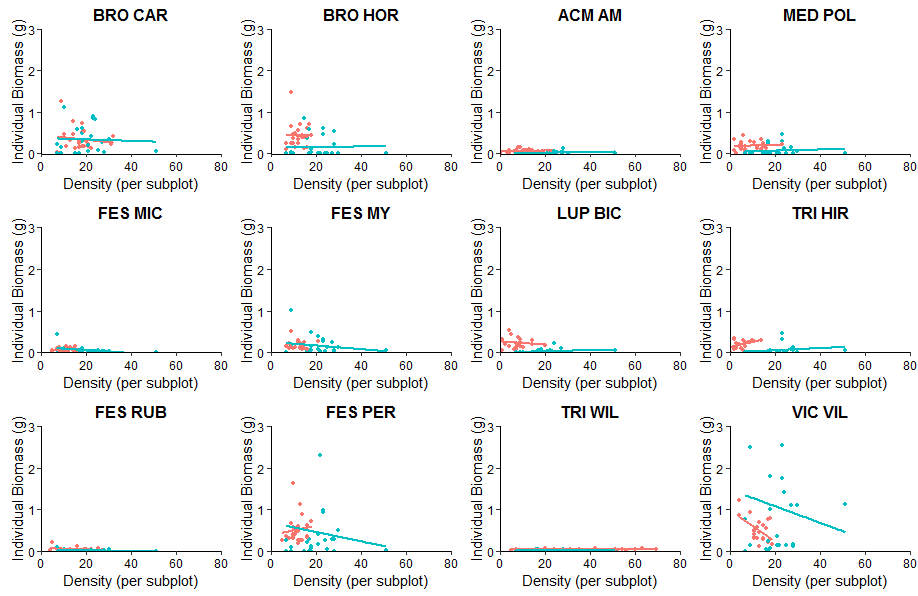
Figure S1. Scatterplots of mean individual biomass in relation to total subplot density for twelve focal species in monoculture subplots (red filled circles) and polyculture subplots (cyan filled circles).


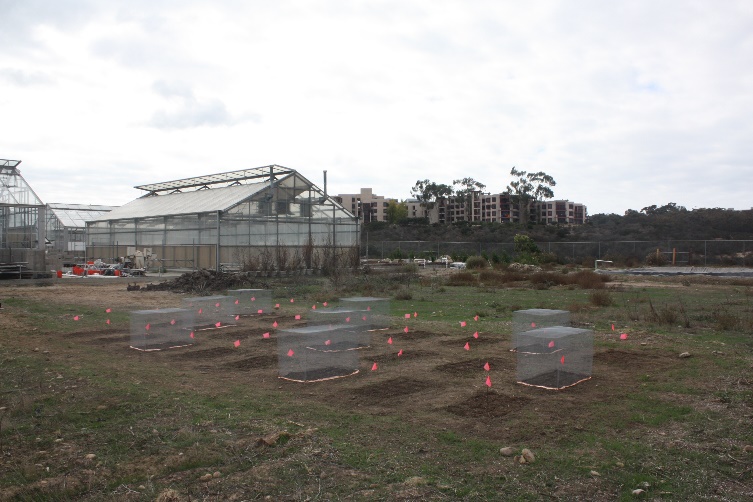
 Figure S2. Photograph of experimental setup at the UC San Diego Biology Field Station.
